# FKBP5 Regulates RIG-I-Mediated NF-κB Activation and Influenza A Virus Infection

**DOI:** 10.3390/v12060672

**Published:** 2020-06-22

**Authors:** Wenzhuo Hao, Lingyan Wang, Shitao Li

**Affiliations:** Department of Microbiology and Immunology, Tulane University, New Orleans, LA 70112, USA; whao2@tulane.edu (W.H.); lwang32@tulane.edu (L.W.)

**Keywords:** IKK, host defense, antiviral, flu, immunophilin

## Abstract

Influenza A virus (IAV) is a highly transmissible respiratory pathogen and is a constant threat to global health with considerable economic and social impact. Influenza viral RNA is sensed by host pattern recognition receptors (PRRs), such as the Toll-like receptor 7 (TLR7) and retinoic acid-inducible gene I (RIG-I). The activation of these PRRs instigates the interferon regulatory factor (IRF) and nuclear factor kappa B (NF-κB) signaling pathways that induce the expression of interferon-stimulated genes (ISGs) and inflammatory genes. FK506-binding protein 5 (FKBP5) has been implied in the IκBα kinase (IKK) complex. However, the role of FKBP5 in the RIG-I signaling and IAV infection is not well elucidated. Here, we demonstrate that the knockout of FKBP5 increases IAV infection. Furthermore, FKBP5 binds IKKα, which is critical for RIG-I-induced innate immune responses and ISG expression. Taken together, FKBP5 is a novel anti-influenza host factor that restricts IAV infection by the activation of RIG-I-mediated NF-κB signaling.

## 1. Introduction

Influenza A virus (IAV) is a negative, single-stranded RNA virus that belongs to the Orthomyxoviridae family. IAV causes seasonal epidemics and has the potential for global pandemics [1,2]. IAV engages with host innate immunity and instigates several innate immune signaling pathways, including TLR7 and RIG-I signaling pathways. RIG-I is a cytosolic RNA sensor that binds IAV RNA. The binding of RNA induces conformational change and several post-translation modifications, such as K63-linked polyubiquitination, which leads to the oligomerization of RIG-I. The oligomerized RIG-I further induces the oligomerization of the mitochondrial antiviral signaling protein (MAVS, also known as CARDIF, IPS1, and VISA). The oligomerized MAVS acts as a signaling platform by recruitment of IKK and TANK-binding kinase 1 (TBK1). IKK and TBK1 activate the transcription factors, NF-κB and IRF, respectively. Then, NF-κB and IRF translocate to the nucleus and form transcriptional complexes to activate type I interferon (IFN) expression. The type I IFN acts as a master cytokine that activates hundreds of interferon-stimulated genes, which in turn inhibit IAV infection.

FKBP5 (also known as FKBP51) belongs to the immunophilin family that consists of FKBP and the tetratricopeptide repeat (TPR) domain. The FKBP domain has peptidylprolyl isomerase (PPIase) activity that catalyzes the *cis*–*trans* conversion of peptidylprolyl bonds, a reaction that is important for protein folding. The TPR domains at the C terminus mediate protein–protein interactions [3,4]. FKBP5 is a well-known molecular chaperone of peptidyl steroid hormone receptors, such as progesterone, androgen, and glucocorticoid receptors [3,4]. In addition, FKBP5 regulates the activity of several kinases, such as AKT1, cyclin-dependent kinase (CDK), and IKK. FKBP5 acts as a scaffolding protein for AKT1 and promotes the dephosphorylation of AKT1, thereby downregulating the AKT signaling pathway [5]. Recent studies found that FKBP5 interacted with CDK4 [6,7]. FKBP5 is required to maintain CDK4 levels in cancer cells [6]. FKBP5 also promotes the *cis*–*trans* isomerization of the Thr172–Pro173 peptide bond in CDK4 and inhibits the phosphorylation of Thr172, an essential step for cell cycle exit and myoblast differentiation [7]. FKBP5 is also found to play a role in NF-κB activation [8,9]. FKBP5 acts as a scaffold by interacting with several components in the tumor necrosis factor alpha (TNFα) pathway, including IKKα, IKKβ, IKKγ, and TNF receptor-associated factor 2 (TRAF2); thus, activating the IKK [9]. However, the role of FKBP5 in the context of RIG-I signaling and IAV infection is not well elucidated.

In this study, we found that the deficiency of FKBP5 in HEK293 and A549 cells increased host susceptibility to IAV infection. Furthermore, FKBP5 preferentially bound IKKα and positively regulated RIG-I-mediated type I IFN activation. Overall, our study suggests that FKBP5 restricts IAV infection by activating RIG-I-mediated NF-κB signaling.

## 2. Materials and Methods

### 2.1. Cells and Viruses

HEK293 cells (American Type Culture Collection, Manassas, VA, USA (ATCC), #CRL-1573) and MDCK cells (ATCC, #CCL-34) were maintained in Dulbecco’s modified Eagle medium (Life Technologies) containing penicillin–streptomycin (Life Technologies) and 10% fetal bovine serum (Life Technologies). A549 cells (ATCC, #CCL-185) were cultured in the Roswell Park Memorial Institute Medium (RPMI) Medium 1640 (Life Technologies) plus 10% fetal bovine serum and 1× MEM non-essential amino acids solution (Life Technologies).

Influenza A/Puerto Rico/8/34 (H1N1) was purchased from Charles River Laboratories, Wilmington, MA, USA (# 10100374). Influenza PR8-GLuc virus was a generous gift from Dr. Peter Palese and featured a Gaussia luciferase (Gluc) gene inserted downstream of PB2 [10]. IAV delNS1 was a gift from Dr. Adolfo Garcia-Sastre (Mount Sinai School of Medicine, New York City, NY, USA). IAV was propagated in specific pathogen-free fertilized eggs Premium Plus (Charles River Laboratories, Wilmington, MA, USA), as described by Szretter et al. [11]. Nine to eleven day old embryonated chicken eggs were used for the production of the influenza virus. In addition, 0.2 mL stock influenza virus at 1 × 10^3^ TCID50 was injected through the puncture hole into the allantoic cavity. After 72 h of incubation, allantoic fluid was collected. IAV titers were determined by plaque assay as described by Matrosovich et al. [12]. Briefly, 1.2 × 10^6^ MDCK cells per ml were split into 6-well plates. After 2× washes with Dulbecco’s modified Eagle medium (DMEM), serial dilutions of IAV were adsorbed onto the cells for 1 h. The cells were covered with DMEM containing 1% Avicel RC591 NF (FMC Biopolymer, Philadelphia, PA, USA) and 1 µg/mL TPCK-trypsin (Thermo Fisher Scientific, Waltham, MA, USA, # 20233). Crystal violet staining was performed 48 h.p.i., and visible plaques were counted.

### 2.2. Constructs and Reagents

FKBP5 was cloned into pCMV-3Tag-8 (Agilent Technologies, Santa Clara, CA, USA, # 240203) to generate FKBP5-FLAG. Deletion mutants of FKBP5-FLAG were constructed using a Q5^®^ Site-Directed Mutagenesis Kit (New England Biolabs, Ipswich, MA, USA). Human IKKα was cloned into pCMV-3Tag-8 to generate IKKα-HA and IKKα-Myc.

Anti-β-actin (Abcam, Cambridge, MA, USA, # ab8227), anti-FLAG (Sigma, St. Louis, MO, USA, # F3165), anti-hemagglutinin (HA) epitope (Cell Signaling Technology, Danvers, MA, USA, # 3724), anti-Myc (Bethyl Laboratories, Montgomery, TX, USA, # A190-105A), anti-interferon-induced transmembrane protein 3 (IFITM3) (GeneTex, Irvine, CA, USA, # GTX63349), anti-FKBP5 (Abcam, # ab126715), anti-non-structural protein 1 (NS1) (Santa Cruz Biotechnology, Dallas, TX, USA, # sc-130568), anti- nucleoprotein (NP) (GenScript, Piscataway, NJ, USA, # A01506-40), anti-IKKα (Cell Signaling Technology, # 11930S) were also obtained, as were goat anti-mouse IgG-horseradish peroxidase (HRP) (Bethyl Laboratories, # A90-116P), and goat anti-rabbit IgG-HRP (Bethyl Laboratories, # A120-201P).

### 2.3. Plasmid Transfection

Transfections using Lipofectamine 2000 or Lipofectamine 3000 transfection reagent (Life Technologies) were performed according to the manufacturer’s protocol. For co-immunoprecipitation (co-IP) experiments, a total of 2.5 µg plasmids was transfected into approximately 1.2 × 10^6^ cells. For the other experiments, a total of 0.5 µg plasmids was transfected into approximately 2 × 10^5^ cells.

### 2.4. Sample Preparation, Western Blotting, and Immunoprecipitation

Approximately 1 × 10^7^ cells were lysed in 500 µL tandem affinity purification (TAP) lysis buffer (50 mM Tris-HCl (pH 7.5), 10 mM MgCl_2_, 100 mM NaCl, 0.5% Nonidet P40, 10% glycerol, Complete Ethylenediaminetetraacetic Acid (EDTA)-free protease inhibitor cocktail tablets (Roche, Indianapolis, IN, USA)) for 30 min on ice. The lysates were then centrifuged for 30 min at 15,000× *g*. The supernatants were collected and mixed with a 1× Lane Marker Reducing Sample Buffer (Thermo Fisher Scientific). Western blotting and immunoprecipitation were performed as described in a previous study [13].

### 2.5. Proteomics Analysis of FKBP5 Protein Complex

Affinity purification coupled with mass spectrometry (AP–MS) experiments were performed as previously described [13]. For the protein purification, HEK293 cells stably expressing FLAG-tagged FKBP5 were collected and lysed in 10 mL of the TAP lysis buffer. Cell lysates were pre-cleared with 50 µL protein A and G resin before the addition of 20 µL of anti-FLAG resin (Sigma, # F2426) and incubated for 16 h at 4 °C on a rotator. The resin was washed three times and transferred to a spin column with 40 µL of the FLAG peptide for 1 h at 4 °C.

The purified samples were sent for mass spectrometry analysis. Proteins found in the control group were considered as non-specific binding proteins. The SAINT algorithm (http://sourceforge.net/projects/saint-apms) was used to evaluate the MS data [14]. Proteins with SAINT score <0.89 or with <3 peptide hits were considered as non-specific binding proteins.

### 2.6. Real-Time PCR

Total RNA was prepared using the RNeasy Mini Kit (Qiagen, Germantown, MD, USA, # 74106). Five hundred nanograms of RNA was reverse transcribed into cDNA using the ProtoScript First Strand cDNA Synthesis Kit (New England Biolabs, # E6300S). For one real-time reaction, 10 µL of SYBR Green PCR reaction mix (Eurogentec, Liège, Belgium) including 0.1 µg of the synthesized cDNA plus an appropriate oligonucleotide primer pair were analyzed on a 7500 Fast Real-time PCR System (Applied Biosystems, Foster City, CA, USA). The comparative cycle threshold (Ct) method was used to determine the relative mRNA expression of genes normalized by the housekeeping gene GAPDH. The primer sequences: human GAPDH, forward primer 5′-AGGTGAAGGTCGGAGTCA-3′, reverse primer 5′-GGTCATTGATGGCAACAA-3′; human CXCL10 (IP10), forward primer 5′-TTCAAGGAGTACCTCTCTCTAG-3′, reverse primer 5′-CTGGATTCAGACATCTCTTCTC-3′; and human CCL5 (RANTES) qPCR primers were purchased from Qiagen (# PPH00703B-200).

### 2.7. RNA Sequencing

Total RNA was prepared using the RNeasy Mini Kit (Qiagen, # 74106). RNA samples were sent to Novogene (Sacramento, CA, USA) for sequencing. Each sample was sequenced to generate a minimum of 20 million reads. The paired-end reads were directionally mapped to the human genome (GRCh38/hg38) using TopHat2. The Cufflink and CuffDiff analyses were performed to identify the differentially expressed genes using a fold change of ≥2 and a false discovery rate (FDR) of <0.05.

### 2.8. CRISPR/Cas9

The single guide RNA (sgRNA) sequence targeting human FKBP5 is 5′-ATCCGGAGAACCAAACGGAA-3′. The sgRNA was cloned into LentiCRISPR v2 [15] (Addgene). Additionally, 0.5 µg of the lentiviral construct was transfected into HEK293 cells using Lipofectamine 2000. The cells were selected with 10 µg/mL puromycin for 14 days. Single clones were expanded for knockout confirmation by Western blotting and DNA sequencing.

### 2.9. Statistical Analysis

The sample size was sufficient for the data analysis using paired two-tailed Student’s *t*-test. For all statistical analyses, the differences were considered to be statistically significant at values of *p* < 0.05.

## 3. Results

### 3.1. Deficiency of FKBP5 Increases Host Susceptibility to IAV Infection

**Figure 1:**
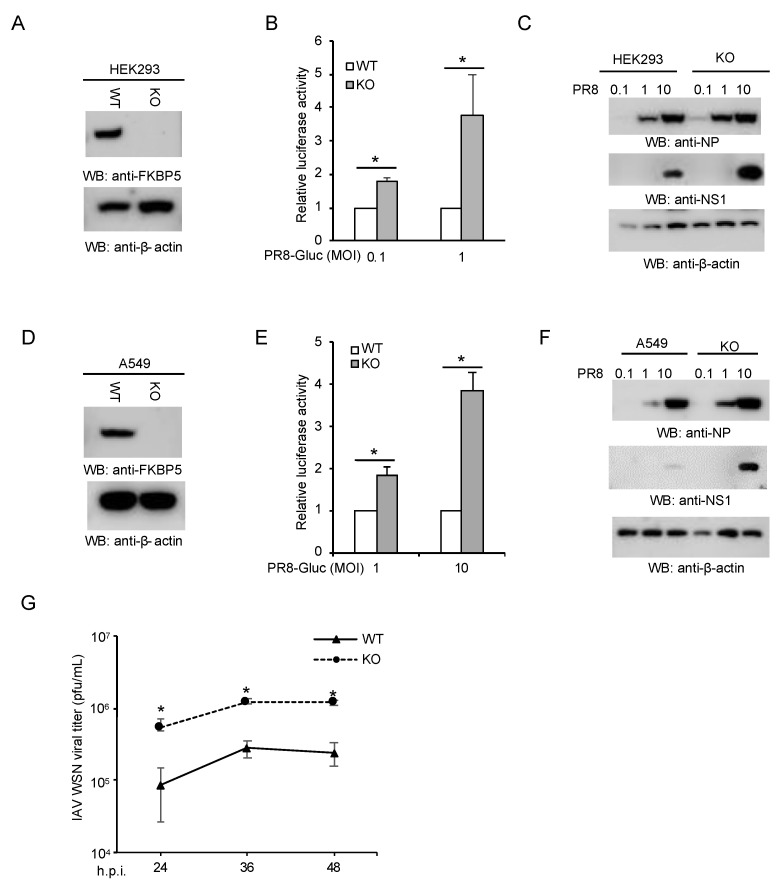
Deficiency of FKBP5 increases host susceptibility to influenza A virus (IAV) infection. (**A**) CRISPR knockout of FKBP5 in HEK293 cells. Cell lysates were blotted as indicated. (**B**) Wild-type (WT) HEK293 and FKBP5 knockout (KO) cells were infected at multiplicity of infection (MOI) of 0.1 or MOI 1 with IAV PR8-Gluc for 24 h. Relative luciferase activities were examined. All experiments were biologically repeated three times. *, *p* < 0.05. (**C**) FKBP5 wild-type and knockout HEK293 cells were infected with the designated MOI of IAV PR8 for 24 h. Cell lysates were blotted using the indicated antibodies. (**D**) CRISPR knockout of FKBP5 in A549 cells. Cell lysates were blotted as indicated. (**E**) FKBP5 wild-type and knockout A549 cells were infected at MOI of 1 or 10 with IAV PR8-Gluc for 24 h. Relative luciferase activities were examined. All experiments were biologically repeated three times. *, *p* < 0.05. (**F**) FKBP5 wild-type and knockout A549 cells were infected with the designated MOI of IAV PR8 for 24 h. Cell lysates were blotted using the indicated antibodies. (**G**) Wild-type and FKBP5 knockout A549 cells were infected at an MOI of 0.1 with influenza A/WSN/1933 (WSN). After the designated hour post-infection (h.p.i.), the virus titers were determined by plaque assay in MDCK cells. All experiments were biologically repeated three times.

To determine the role of FKBP5 in IAV infection, we first depleted FKBP5 in HEK293 cells by CRISPR. In this regard, a sgRNA was cloned into the lentiCRISPR v2 containing Cas9 and transfected into HEK293 cells. After 48 h, the cells were selected using puromycin. Single clones were picked and expanded for knockout confirmation by Western blotting (WB). FKBP5 expression was abolished in CRISPR knockout cells as shown by WB (Figure 1A). Next, we infected FKBP5 wild-type and knockout HEK293 cells with a PR8-Gaussia luciferase reporter virus, PR8-Gluc [10]. The reporter assay showed that the PR8-GLuc reporter activity increased in FKBP5 knockout cells compared to the wild-type controls (Figure 1B). In line with these results, the protein expression levels of NS1 and NP were significantly higher in knockout cells (Figure 1C). To corroborate our observations in HEK293 cells, we further knocked out FKBP5 in human lung epithelial cells A549 by CRISPR (Figure 1D). Similarly, the deficiency of FKBP5 increased PR8-Gluc reporter activity (Figure 1E) and viral protein expression levels (Figure 1F). We also evaluated the impact of FKBP5 deficiency on the viral propagation of WSN IAV using the plaque assay. As predicted, FKBP5 knockout cells produced more infectious viral particles than the control A549 cells (Figure 1G). Taken together, these data suggest that endogenous FKBP5 is essential for host defense to IAV.

### 3.2. FKBP5 Is Not an Interferon-Stimulated Gene

**Figure 2:**
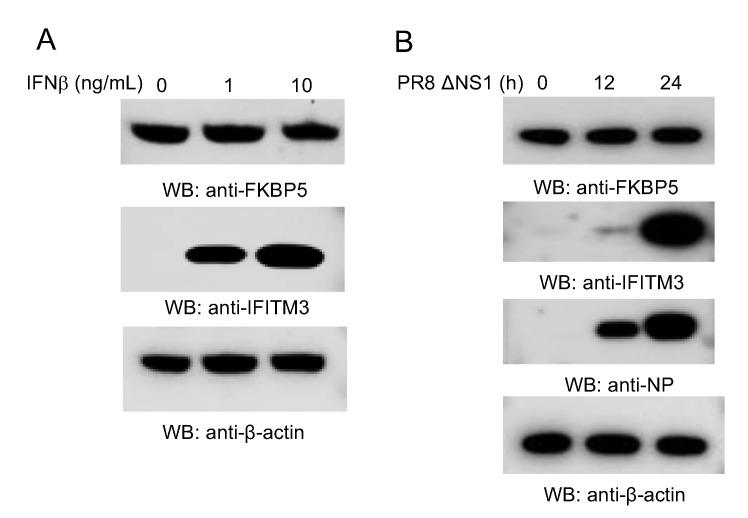
FKBP5 expression is not modulated by IFN and IAV. (**A**) A549 cells were treated with the designated amount of IFNβ for 12 h. Cell lysates were blotted with the indicated antibodies. The interferon-induced transmembrane protein 3 (IFITM3) was included as a positive control. (**B**) A549 cells were infected with 1 MOI of PR8 ΔNS1 IAV for 0 h, 12 h, and 24 h. Cell lysates were blotted with the indicated antibodies.

As IAV is intrinsically sensitive to IFN, we speculated that type I IFN might regulate FKBP5 expression. Therefore, we treated A549 cells with IFN-β or infected with a PR8 IAV mutant with an NS1 gene deletion (PR8 ΔNS1). FKBP5 protein expressions were then examined. However, IFN-β stimulation or IAV infection marginally modulated FKBP5 protein levels (Figure 2A,B), suggesting that FKBP5 is constitutively expressed in A549 cells.

### 3.3. FKBP5 Interacts with IKKα

**Figure 3:**
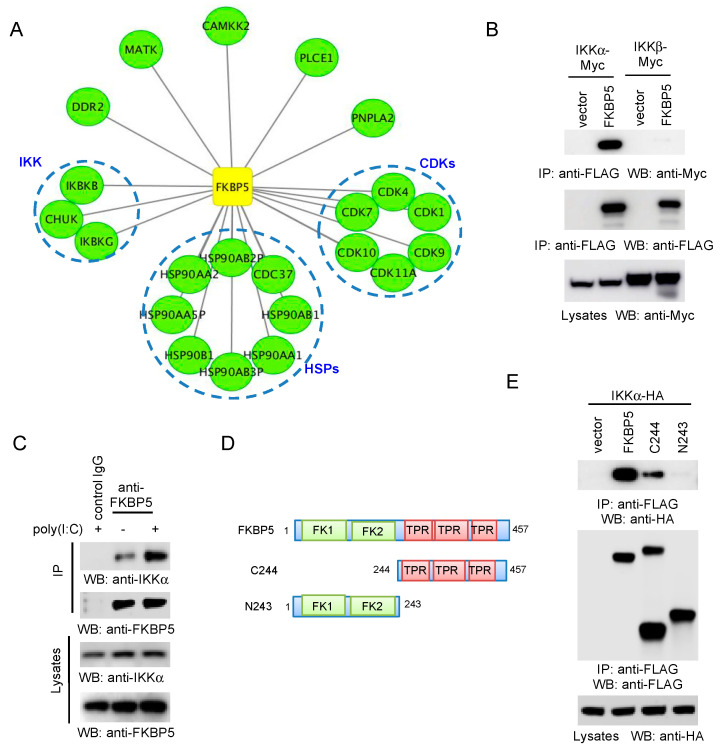
FKBP5 interacts with IKKα. (**A**) Map of the FKBP5 protein interaction network. FKBP5 and the high confidence candidate interacting proteins (HCIPs) are shown as square and circles, respectively. (**B**) Vector or FLAG-tagged FKBP5 was co-transfected with Myc-tagged IKKα or IKKβ into HEK293 cells. After 48 h, cell lysates were immunoprecipitated and blotted as indicated. (**C**) HEK 293 cells were stimulated with 1 µg/mL poly(I:C) for 4 h. Cell lysates were immunoprecipitated and blotted as indicated. The same isotype Immunoglobulin G (IgG) was used as a control for immunoprecipitation (IP). (**D**) Schematics of the FKBP5 mutants. FK stands for FKBP; TPR stands for tetratricopeptide repeats. (**E**) Hemagglutinin (HA)-tagged IKKα was transfected with the pCMV-3Tag-8 vector, FLAG-tagged FKBP5, or the indicated FLAG-tagged FKBP5 mutant. After 48 h, the cell lysates were immunoprecipitated and blotted as indicated.

To discover new FKBP5-interacting proteins, we performed the AP–MS analysis of the FKBP5 protein complex. First, FLAG-tagged FKBP5 was transfected into HEK293 cells to generate a stable cell line. After the stable cell line was established by puromycin selection, FKBP5 protein complexes were purified by affinity purification using the anti-FLAG antibody and were then analyzed by mass spectrometry. The AP–MS was biologically repeated twice. To efficiently reduce false positives in AP–MS, we adopted the well established statistical method SAINT [14]. Using a stringent statistical SAINT score cutoff of 0.89 (*p* < 0.01), we identified 22 high-confidence candidate interacting proteins (HCIPs), including the IKK complex, CDKs and heat shock proteins (HSPs) (Figure 3A).

IKK has been shown to interact with FKBP5; however, whether the endogenous FKBP5 interacts with IKK is not clear. First, we performed co-IP between FKBP5 and IKKα or IKKβ. In this regard, FLAG-tagged FKBP5 was co-transfected with Myc-tagged IKK into HEK293 cells. Interestingly, co-IP found that FKBP5 preferentially interacted with IKKα (Figure 3B). Furthermore, we examined the endogenous protein interaction between FKBP5 and IKKα. We stimulated HEK 293 cells with the viral RNA mimics, poly(I:C), and then performed IP using an anti-FKBP5 antibody. As shown in Figure 3C, endogenous FKBP5 interacted with the endogenous IKKα. Furthermore, poly(I:C) enhanced the IKKα–FKBP5 interaction. Lastly, we determined which FKBP5 domain (Figure 3D) was required for the IKKα interaction. Co-IP demonstrated that the TPR domain was sufficient for the interaction with IKKα (Figure 3E).

### 3.4. FKBP5 Is Required for RIG-I-Induced NF-κB Activity

**Figure 4:**
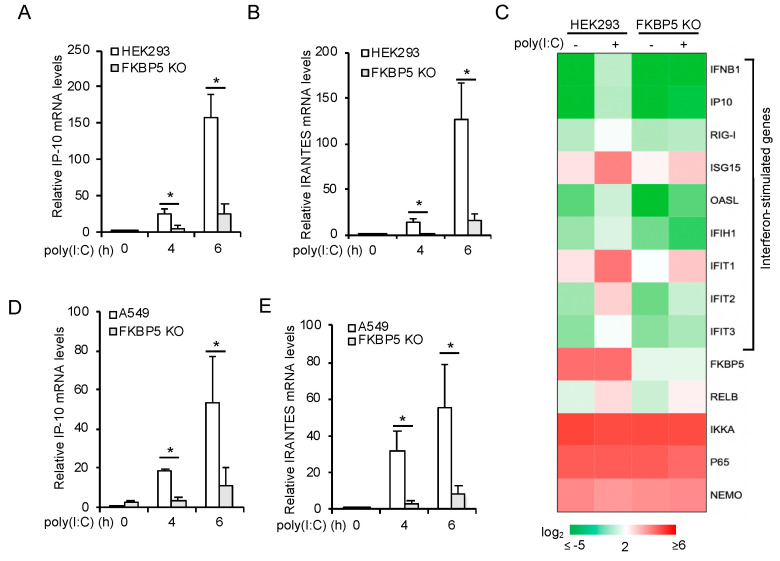
FKBP5 is required for RIG-I signaling. (**A**,**B**) FKBP5 wild-type and knockout HEK293 cells were stimulated with 1 µg/mL poly(I:C) for the indicated times. Real-time PCR was performed to determine the relative mRNA levels of IP-10 (**A**) and RANTES (**B**). All the experiments were biologically repeated three times. Data represent the means ± S.D. of three independent experiments. The *p* value was calculated (two-tailed Student’s *t*-test) by comparison with wild-type cells. An asterisk indicates *p* < 0.05. (**C**) Heatmap of the RNA sequencing results of wild-type and FKBP5 knockout HEK293 cells stimulated with 1 µg/mL poly(I:C) for 4 h. (**D**,**E**) FKBP5 wild-type and knockout A549 cells were stimulated with 1 µg/mL poly(I:C) for the indicated times. Real-time PCR was performed to determine the relative mRNA levels of IP-10 (**D**) and RANTES (**E**). All the experiments were biologically repeated three times. Data represent the means ± S.D. of three independent experiments. The *p* value was calculated (two-tailed Student’s *t*-test) by comparison with wild-type cells. An asterisk indicates *p* < 0.05.

RIG-I is a sensor to IAV RNA and activates the IKK and TBK1 signaling pathways to induce type I IFN expression. FKBP5 has been shown to be required for IKK activity; however, to our knowledge, whether FKBP5 is critical for RIG-I–IKK signal axis is unknown. Thus, we first stimulated the FKBP5 wild-type and knockout HEK 293 cells with poly(I:C) and examined the mRNA expression of two ISGs, IP-10 and RANTES. Real-time PCR assays showed that FKBP5 deficiency impaired the poly(I:C)-induced mRNA expression of IP-10 (Figure 4A) and RANTES (Figure 4B). RNA sequencing further revealed that the mRNA expression of IFNβ and several ISGs were reduced in FKBP5 knockout cells (Figure 4C). To complement the results in FKBP5 knockout HEK293 cells, we also examined the effects of FKBP5 knockout in A549 cells. Consistently, poly(I:C)-induced mRNA expression of IP-10 (Figure 4D) and RANTES (Figure 4E) was also impaired in FKBP5 knockout A549 cells. Overall, these data suggest that FKBP5 is required for the RIG-I signaling pathway.

## 4. Discussion

The pattern recognition receptors (PRRs) are the sentinels of the innate immune system, which discriminate the microbial components distinct from the host [16]. RIG-I is a cytosolic PRR and recognizes double-stranded RNA (dsRNA) or 5′ triphosphate RNA in the cytoplasm generated by RNA viruses. Upon the engagement with viral RNA, RIG-I activates signaling cascades that lead to the induction of type I IFN expression [17,18,19,20,21]. Type I IFN activates the mRNA expression of hundreds of ISGs, Most of the ISGs are antiviral proteins, such as IFITM3 and Viperin. In addition to ISGs, a group of antiviral host factors, termed intrinsic immunity factors, are constitutively expressed and IFN-independent. The “pre-existed” expression of these intrinsic host factors guarantees a more rapid response and a direct inhibition of viral infection [22,23]. For example, TRIM41 targets the nucleoproteins of IAV and vesicular stomatitis virus (VSV) for K48-linked polyubiquitination and protein degradation, thereby impeding IAV and VSV infection [24,25]. Here, we report that FKBP5 participates in RIG-I-mediated NF-κB signal cascade that is essential for type I IFN expression.

Our study suggests that FKBP5 regulates NF-κB via the IKK complex. However, FKBP5 might also modulate NF-κB activity indirectly through steroid receptors. For example, glucocorticoids are potent anti-inflammatory agents by activating glucocorticoid receptor (GR) [26]. A study showed that FKBP5 cooperated with another immunophilin FKBP4 (also known as FKBP52) to modulate GR-mediated NF-κB suppression [27]. The overexpression of FKBP5 inhibits NF-κB nuclear translocation and NF-κB transcriptional activity. By contrast, FKBP4 counteracts the effects of FKBP5 [27]. Although their conclusion is mainly based on overexpression, it suggests that the optimal expression of FKBP5 might be required for NF-κB activation.

Many genes involved in innate immunity are ISGs; however, we found that FKBP5 expression was not induced by IFN and IAV in A549 cells. Our data suggest that FKBP5 is constitutively expressed. A recent study found that the expression of FKBP5 was induced by glucocorticoid in A549 cells, which facilitates the suppressive effect of glucocorticoid on pro-inflammatory cytokine production [28]. Further studies will investigate the differential role of FKBP5 in RIG-I and glucocorticoid pathways.

The NS1 of IAV inhibits host innate immune defenses through diverse mechanisms [22]. First, NS1 inhibits the cellular double-stranded RNA sensors, including RIG-I, protein kinase R, and 2′-5′ oligoadenylate synthetase. Furthermore, NS1 blocks the regulators of these sensors, such as PRKRA, TRIM25 and NF90. A recent study also found that NS1 bound the kinase domain of IKKα and IKKβ and impaired their kinase activity, thereby inhibiting NF-κB activity [29]. However, whether NS1 antagonizes the function of FKBP5 is unknown. Future studies will investigate the interactions between NS1 and FKBP5.

On the host side, the erratic activation of NF-κB is detrimental due to the overexpression of proinflammatory factors. Thus, feedback control of NF-κB activity is essential. Recently, the IFN-induced protein 44 (IFI44) was found to negatively modulate the NF-κB activity induced after viral infections [30]. Interestingly, IFI44 inhibits IKK kinase activity through binding FKBP5. It is worthy to note that NF-κB activity is also required for the efficient viral replication of IAV [31,32]. The blocking of NF-κB activity using inhibitors impaired the IAV infection in cells [33]. However, what NF-κΒ-dependent genes are activated, how they are specifically activated by the virus, and how they facilitate viral infection are still not well elucidated. Nonetheless, the virus knows how to skew the balance of NF-κB activity to achieve the ideal environment for viral replication, and at the same time suppress host innate immune responses. Future work will pinpoint the mechanisms of how IAV gains the control to precisely induce the NF-κΒ-dependent genes required for viral infection.

In conclusion, FKBP5 is not only required for the RIG-I–IKK signaling axis for type I IFN production, but also inhibits IAV infection. Our study suggests that FKBP5 is a novel anti-influenza gene. Thus, our study provides a foundation for the future development of antiviral therapeutics.

## References

[1] Tscherne D.M., Garcia-Sastre A. (2011). Virulence determinants of pandemic influenza viruses. J. Clin. Investig..

[2] Fukuyama S., Kawaoka Y. (2011). The pathogenesis of influenza virus infections: The contributions of virus and host factors. Curr. Opin. Immunol..

[3] Li L., Lou Z., Wang L. (2011). The role of FKBP5 in cancer aetiology and chemoresistance. Br. J. Cancer.

[4] Storer C.L., Dickey C.A., Galigniana M.D., Rein T., Cox M.B. (2011). FKBP51 and FKBP52 in signaling and disease. Trends Endocrinol. Metab..

[5] Pei H., Li L., Fridley B.L., Jenkins G.D., Kalari K.R., Lingle W., Petersen G., Lou Z., Wang L. (2009). FKBP51 affects cancer cell response to chemotherapy by negatively regulating Akt. Cancer Cell.

[6] Jirawatnotai S., Sharma S., Michowski W., Suktitipat B., Geng Y., Quackenbush J., Elias J.E., Gygi S.P., Wang Y.E., Sicinski P. (2014). The cyclin D1-CDK4 oncogenic interactome enables identification of potential novel oncogenes and clinical prognosis. Cell Cycle.

[7] Ruiz-Estevez M., Staats J., Paatela E., Munson D., Katoku-Kikyo N., Yuan C., Asakura Y., Hostager R., Kobayashi H., Asakura A. (2018). Promotion of Myoblast Differentiation by Fkbp5 via Cdk4 Isomerization. Cell Rep..

[8] Bouwmeester T., Bauch A., Ruffner H., Angrand P.O., Bergamini G., Croughton K., Cruciat C., Eberhard D., Gagneur J., Ghidelli S. (2004). A physical and functional map of the human TNF-alpha/NF-kappa B signal transduction pathway. Nat. Cell Biol..

[9] Romano S., Xiao Y., Nakaya M., D’Angelillo A., Chang M., Jin J., Hausch F., Masullo M., Feng X., Romano M.F. (2015). FKBP51 employs both scaffold and isomerase functions to promote NF-kappaB activation in melanoma. Nucleic Acids Res..

[10] Heaton N.S., Leyva-Grado V.H., Tan G.S., Eggink D., Hai R., Palese P. (2013). In Vivo Bioluminescent Imaging of Influenza A Virus Infection and Characterization of Novel Cross-Protective Monoclonal Antibodies. J. Virol..

[11] Szretter K.J., Balish A.L., Katz J.M. (2006). Influenza: Propagation, quantification, and storage. Curr. Protoc. Microbiol..

[12] Matrosovich M., Matrosovich T., Garten W., Klenk H.D. (2006). New low-viscosity overlay medium for viral plaque assays. Virol. J..

[13] Wang L., Fu B., Li W., Patil G., Liu L., Dorf M.E., Li S. (2017). Comparative influenza protein interactomes identify the role of plakophilin 2 in virus restriction. Nat. Commun..

[14] Choi H., Larsen B., Lin Z.Y., Breitkreutz A., Mellacheruvu D., Fermin D., Qin Z.S., Tyers M., Gingras A.C., Nesvizhskii A.I. (2011). SAINT: Probabilistic scoring of affinity purification-mass spectrometry data. Nat. Methods.

[15] Sanjana N.E., Shalem O., Zhang F. (2014). Improved vectors and genome-wide libraries for CRISPR screening. Nat. Methods.

[16] Takeuchi O., Akira S. (2010). Pattern recognition receptors and inflammation. Cell.

[17] Takeuchi O., Akira S. (2009). Innate immunity to virus infection. Immunol. Rev..

[18] Sharma S., tenOever B.R., Grandvaux N., Zhou G.P., Lin R., Hiscott J. (2003). Triggering the interferon antiviral response through an IKK-related pathway. Science.

[19] Fitzgerald K.A., McWhirter S.M., Faia K.L., Rowe D.C., Latz E., Golenbock D.T., Coyle A.J., Liao S.M., Maniatis T. (2003). IKKepsilon and TBK1 are essential components of the IRF3 signaling pathway. Nat. Immunol..

[20] Hemmi H., Takeuchi O., Sato S., Yamamoto M., Kaisho T., Sanjo H., Kawai T., Hoshino K., Takeda K., Akira S. (2004). The roles of two IkappaB kinase-related kinases in lipopolysaccharide and double stranded RNA signaling and viral infection. J. Exp. Med..

[21] McWhirter S.M., Fitzgerald K.A., Rosains J., Rowe D.C., Golenbock D.T., Maniatis T. (2004). IFN-regulatory factor 3-dependent gene expression is defective in Tbk1-deficient mouse embryonic fibroblasts. Proc. Natl. Acad. Sci. USA.

[22] Zhao M., Wang L., Li S. (2017). Influenza A Virus-Host Protein Interactions Control Viral Pathogenesis. Int. J. Mol. Sci..

[23] Patil G., Li S. (2019). Tripartite motif proteins: An emerging antiviral protein family. Future Virol..

[24] Patil G., Zhao M., Song K., Hao W., Bouchereau D., Wang L., Li S. (2018). TRIM41-Mediated Ubiquitination of Nucleoprotein Limits Influenza A Virus Infection. J. Virol..

[25] Patil G., Xu L., Wu Y., Song K., Hao W., Hua F., Wang L., Li S. (2020). TRIM41-Mediated Ubiquitination of Nucleoprotein Limits Vesicular Stomatitis Virus Infection. Viruses.

[26] Nelson G., Wilde G.J., Spiller D.G., Kennedy S.M., Ray D.W., Sullivan E., Unitt J.F., White M.R. (2003). NF-kappaB signalling is inhibited by glucocorticoid receptor and STAT6 via distinct mechanisms. J. Cell Sci..

[27] Erlejman A.G., De Leo S.A., Mazaira G.I., Molinari A.M., Camisay M.F., Fontana V., Cox M.B., Piwien-Pilipuk G., Galigniana M.D. (2014). NF-kappaB transcriptional activity is modulated by FK506-binding proteins FKBP51 and FKBP52: A role for peptidyl-prolyl isomerase activity. J. Biol. Chem..

[28] Chan P.K. (2010). The Role of FKBP5 in Influenza Virus Infection. Master’s Thesis.

[29] Gao S., Song L., Li J., Zhang Z., Peng H., Jiang W., Wang Q., Kang T., Chen S., Huang W. (2012). Influenza A virus-encoded NS1 virulence factor protein inhibits innate immune response by targeting IKK. Cell. Microbiol..

[30] DeDiego M.L., Nogales A., Martinez-Sobrido L., Topham D.J. (2019). Interferon-Induced Protein 44 Interacts with Cellular FK506-Binding Protein 5, Negatively Regulates Host Antiviral Responses, and Supports Virus Replication. mBio.

[31] Kumar N., Xin Z.T., Liang Y., Ly H., Liang Y. (2008). NF-kappaB signaling differentially regulates influenza virus RNA synthesis. J. Virol..

[32] Wurzer W.J., Ehrhardt C., Pleschka S., Berberich-Siebelt F., Wolff T., Walczak H., Planz O., Ludwig S. (2004). NF-kappaB-dependent induction of tumor necrosis factor-related apoptosis-inducing ligand (TRAIL) and Fas/FasL is crucial for efficient influenza virus propagation. J. Biol. Chem..

[33] Nimmerjahn F., Dudziak D., Dirmeier U., Hobom G., Riedel A., Schlee M., Staudt L.M., Rosenwald A., Behrends U., Bornkamm G.W. (2004). Active NF-kappaB signalling is a prerequisite for influenza virus infection. J. Gen. Virol..

